# C-954-17. Where Architecture Meets Burn Units: AI-Driven Material Selection Approaches to Enhance Infection Control

**DOI:** 10.1093/jbcr/irag033.149

**Published:** 2026-04-06

**Authors:** Alyssa D Brown, Alan Pang, Nero Chenxuan He, Lingyi Qiu

**Affiliations:** Texas Tech University Health Sciences Center, Lubbock, TX; Texas Tech University Health Sciences Center, Lubbock, TX; Texas Tech University Health Sciences Center, Lubbock, TX; Texas Tech University Health Sciences Center, Lubbock, TX

## Abstract

**Introduction:**

Infection control is often managed primarily by health professionals through medical equipment or operational protocols. The architectural design of healthcare facilities can provide a stable base upon which to establish the spatial, material, and infrastructural conditions upon which medical protocols can operate more successfully. Importantly, material selection represents a highly effective and practical strategy for infection control. While it is easier to implement small scale strategies to prevent infection, in the long run, full-unit remodeling, infrastructural replacement, or spatial re-planning can yield disproportionately significant outcomes in reducing the spread and occurrence of nosocomial infections. Infection is the leading cause of morbidity and mortality in burn patients, and burn centers are at high risk for nosocomial infection. Architectural interventions can be an important option for limiting infections.

**Methods:**

This interdisciplinary study is based on a Level 1 Burn Center at an academic hospital. A combination of 3D scanning and AI-augmented metadata structuring was employed to document, evaluate, and identify material properties that are critical to infection control. The methodology further included a systematic assessment of interior spatial materials in relation to their infection-control performance.

**Results:**

Some materials are more porous than others leading to decreased penetration of antimicrobial solutions. The design of tank rooms are even more important in that they are so frequently used and by different patients during the day. During an outbreak of Carbapenem resistant acinitobacter braumanni, it was found that adding filters to the faucets of the sink vastly decreased spread of the bacteria.

**Conclusions:**

Burn units have unique needs both in terms of patients and architecture. Infections are a danger to the morbidity and mortality of burn patients. Analyzing existing materials to help control infections. The study provides an evaluation of existing materials in the burn center with respect to infection control and proposes optimized strategies for material selection and replacement to enhance performance.

**Applicability of Research to Practice:**

These findings can inform future healthcare planning and facility design, while also supporting improved infection-control operations in specialized medical environments.

**Funding for the study:**

N/A.

